# Maternal Gut Microbiota in Gestational Diabetes Mellitus and Fetal Macrosomia: Is There an Association?

**DOI:** 10.3390/biomedicines13122941

**Published:** 2025-11-29

**Authors:** Lejla Pašić, Katja Molan, Draženka Pongrac Barlovič, Marjanca Starčič Erjavec, Darja Žgur Bertok, Jerneja Ambrožič Avguštin

**Affiliations:** 1 Sarajevo Medical School, Sarajevo School of Science and Technology, Hrasnička cesta 3a, 71000 Sarajevo, Bosnia and Herzegovina; lejla.pasic@ssst.edu.ba; 2 Faculty of Health Sciences, University of Novo mesto, Na Loko 2, 8000 Novo mesto, Slovenia; katja.molan@uni-nm.si; 3 Clinical Department of Endocrinology, Diabetes and Metabolic Diseases, University Medical Centre Ljubljana, Zaloška 7, 1000 Ljubljana, Slovenia; drazenka.pongrac@gmail.com; 4 Medical Faculty, University of Ljubljana, Vrazov trg 2, 1000 Ljubljana, Slovenia; 5 Department of Microbiology, Biotechnical Faculty, University of Ljubljana, Večna pot 111, 1000 Ljubljana, Slovenia; marjanca.starcic.erjavec@bf.uni-lj.si; 6 Department of Biology, Faculty of Natural Sciences and Mathematics, University of Maribor, Koroška cesta 160, 2000 Maribor, Slovenia; 7 Department of Biology, Biotechnical Faculty, University of Ljubljana, Večna pot 111, 1000 Ljubljana, Slovenia; darja.zgur@bf.uni-lj.si

**Keywords:** gut microbiota, gestational diabetes, large for gestational age infants, gestational weight gain

## Abstract

**Background/Objectives**: Gestational diabetes mellitus (GDM) is associated with altered maternal gut microbiota and increased risk of large-for-gestational age (LGA) births. The contribution of gut microbiota to fetal overgrowth in GDM, independent of glycemic control, remains unclear. **Methods**: In this pilot longitudinal study, the gut microbiota of 18 women with GDM was followed from the second (2T) to the third trimester (3T). Maternal fecal samples were analyzed by 16S rRNA gene sequencing, and associations between microbial profiles and infant birth weight were examined. In addition, these associations were adjusted for pre-pregnancy body mass index (BMI) and gestational weight gain (GWG). **Results**: Maternal gut microbiota of LGA infants exhibited consistently lower microbial diversity, a reduced Bacillota/Bacteroidota ratio, and enrichment of pro-inflammatory taxa including *Prevotella*, *Sutterella*, and *Bilophila*. Short-chain fatty acids (SCFAs)-producing genera such as *Acinetobacter*, *Odoribacter*, *Faecalibacterium*, and *Lachnoclostridium* were depleted. Although *Bilophila* was identified as a third-trimester biomarker with LEfSE approach, its association with LGA disappeared after adjusting for BMI and GWG. Conversely, Nitrospirota, *Polaromonas*, *Acinetobacter*, and *Aeromonas* correlated negatively with LGA even after BMI and GWG adjustment. **Conclusions**: These findings suggest that specific maternal microbiota signatures, together with pre-pregnancy adiposity, influence fetal overgrowth in GDM and may serve as early biomarkers or targets for preventive interventions.

## 1. Introduction

Gestational diabetes mellitus (GDM) is defined by the World Health Organization as hyperglycemia first detected in the second (2T) or third trimester (3T) of pregnancy, with glucose levels below the threshold for overt diabetes [1]. Globally, GDM affects an estimated 9–27% of pregnancies, and its prevalence continues to rise in parallel with sedentary lifestyles, rising maternal age, and dietary transitions [2]. GDM substantially increases the risk of adverse outcomes for both mother and child, including hypertensive disorders of pregnancy, preterm birth, and long-term cardiometabolic disease [3].

A prominent and clinically important complication of GDM is excessive fetal growth, which is characterized as large-for-gestational-age (LGA) or macrosomia [4,5]. LGA is typically defined as birthweight above the 90th percentile for gestational age and sex [6] and macrosomia as birthweight exceeding 4000 g [7]. Up to 45% of infants born to mothers with GDM meet the above criteria, compared with only ~12% among infants of normoglycemic mothers [4]. Excessive fetal growth increases risks for delivery complications and long-term metabolic disorders [7,8,9].

Fetal growth is shaped by multiple maternal and fetal determinants. Established maternal risk factors include higher pre-pregnancy body mass index (BMI), excessive gestational weight gain (GWG), older maternal age, and placental dysfunction [10,11,12]. Maternal hyperglycemia is a key driver of accelerated fetal growth via fetal hyperinsulinemia driven by excess glucose and lipids [13,14]. Yet, in many pregnancies, excessive fetal growth occurs even with apparently good glycemic control, indicating that additional mechanisms are likely involved [4,15].

Gut microbes contribute to nutrient processing, bile acid transformation, and immune signaling through the production of bioactive metabolites. Perturbations of gut microbiota have been linked to several conditions, including obesity and type 2 diabetes [16,17]. Accumulating evidence implicates maternal gut microbiota in the development of GDM. Several studies report distinct fecal microbiome signatures in women with GDM compared to normoglycemic pregnancies [18,19,20].

Koren et al. [21] associated increased insulin resistance in the 3T of pregnancy with an increase in Pseudomonadota and Actinomycetota, a decrease in butyrate-producing bacteria, and an increase in the abundance of 18 other taxa, including *Enterobacteriaceae* and *Streptococcus*. Additionally, this study reported a decrease in alpha diversity within the individual gut microbiome and an increase in beta diversity in the gut microbiomes of GDM women.

Mokkala et al. [22] described the gut microbiome in the first trimester of pregnancy and found significantly different abundance of *Ruminococcaceae* in women who later developed GDM compared to normoglycemic women. This difference was positively correlated with maternal glucose levels.

Kuang et al. [23] compared the gut microbiome in GDM and normoglycemic pregnancies during the late second and early third trimesters. They found an increase in the abundance of *Parabacteroides*, *Klebsiella*, and *Catenibacterium* species, and a decrease in butyrate-producing members.

Ferrocino et al. [24] followed GDM pregnancies between the 2T and 3T, reporting an increase in alpha and beta diversity. In terms of phyla abundance, an increase in Bacillota and Pseudomonadota and a decrease in Bacteroidota were found. At genus level, a decrease in *Collinsella* and Rikenellaceae, alongside increased abundance of several butyrate-producing and Firmicutes-associated genera, was reported. This suggests that the alterations in microbiota composition in GDM pregnancies may play a role in the GDM progression.

Crusell et al. [18] noted that the GDM gut microbiota was aberrant at both phylum (increased Actinomycetota) and genus (increased *Collinsella*, *Rothia*, and *Desulfovibrio*) levels compared to the gut microbiota in normoglycemic pregnancies. The GDM gut microbiota resembled that of type 2 diabetes patients, and aberrations were found to persist eight months after delivery.

Finally, Pinto et al. [20] reported that gut microbiome/metabolome profiling and assessment of inflammatory markers during the first trimester could predict GDM before clinical onset in the studied cohort.

Although several studies have characterized microbial alterations in GDM, little is known about the gut microbiota’s role in fetal growth outcomes within GDM pregnancies. Only two previous studies—both in non-GDM populations—have directly associated maternal gut microbiota with fetal overgrowth [8,25]. Both were cross-sectional and sampled microbiota at a single time point, leaving open key questions about longitudinal changes and mechanistic links to excessive fetal growth.

To address this knowledge gap, we conducted a pilot prospective longitudinal study of women with GDM to investigate whether changes in maternal gut microbiota between the 2T and 3T—after dietary counseling and during continued glucose monitoring—are associated with LGA birth outcomes. Specifically, we aimed to (1) characterize shifts in gut microbiota diversity and composition across 2T and 3T in GDM pregnancies; (2) identify bacterial taxa associated with infant birthweight category (LGA vs. NBW), and (3) assess whether these associations are independent of major maternal metabolic factors, particularly pre-pregnancy BMI and GWG.

Our study provides new insights into how maternal gut microbiota, metabolic status, and fetal growth are interconnected. Understanding these relationships could inform early biomarkers and microbiota-targeted interventions, for example, precision probiotics or dietary strategies—to reduce the risk of LGA in GDM pregnancies.

## 2. Materials and Methods

### 2.1. Participant Recruitment

Thirty-nine pregnant women with GDM were consecutively recruited from the outpatient clinic of the University Medical Centre Ljubljana.

### 2.2. Inclusion and Exclusion Criteria

Inclusion criteria were a diagnosis of GDM according to the International Association of Diabetes and Pregnancy Study Groups (IADPSG) criteria [26] during the 2T of pregnancy, and willingness to participate. Women who had received antibiotic therapy within six months of study entry were excluded. Dietary habits before and during pregnancy were assessed via structured questionnaires [27]. In addition, all enrolled pregnant women were non-smokers, according to self-report.

Of the 39 women initially enrolled, 21 were excluded for the following reasons: antibiotic use during pregnancy (*n* = 3), insufficient fecal sample collection in the 2T or 3T (*n* = 9), infants with gestational weight below the 30th percentile (*n* = 7), stillbirth (*n* = 1), and low-quality DNA sequences (*n* = 1). The final analysis, therefore, included 18 women with paired 2T- and 3T microbiota data.

### 2.3. Clinical Management and Sample Collection

A detailed personal history was taken, with collection of demographic variables such as age, level of education, living environment, marital status, employment status (employed/unemployed), and parity. Participants were followed until delivery, and infant birth weights were recorded and adjusted for gestational age and sex. Clinical examination was performed, including basic anthropometric measurements, such as body weight, height, body mass index (BMI), blood pressure, and heart rate [27].

All participants received nutritional counseling immediately after a diagnosis of GDM and were instructed to self-monitor their capillary blood glucose levels in both the fasting state and postprandially. Hyperglycemia was managed with non-pharmacological interventions or insulin when indicated, following national guidelines.

A spectrum of laboratory analyses was performed. Maternal fasting blood, stool samples, and anthropometric measurements were collected at 24–28 weeks of gestation (2T) and again at 38 weeks (3T). Biochemical blood analysis included glucose, HbA1c, insulin, and lipid profiles. Insulin resistance was estimated using the HOMA-IR index, calculated as fasting insulin (μU/L) × fasting glucose (mmol/L)/22.5. Fasting blood glucose concentration was analyzed with an automatic Avria LabCell-Siemens system. Serum insulin and C-peptide were assayed using an enzyme-labeled chemiluminescent immunometric assay (Siemens Healthcare Diagnostics, Deerfield, IL, USA) and were expressed in mU/L and nmol/L, respectively. The following were the references laboratory values: serum insulin concentration (fasting state) of 2–17.2 mU/L; serum C-peptide concentration (fasting state) of 0.3–2.4 nmol/L. Glycated hemoglobin was measured using high-performance liquid chromatography with a BioRad D-100 (Bio-Rad Laboratories, Hercules, CA, USA) automatic analyzer.

For the metataxonomic analysis, participants collected approximately 10 g of feces into sterile containers. The samples were stored under refrigeration and transported on ice to the laboratory within 48 h. Samples were aliquoted (200 mg) and stored at −80 °C without buffer until analysis.

### 2.4. DNA Extraction and 16S rRNA Gene Sequencing

Total microbial DNA was extracted from 200 mg of feces using the PS Spin Stool DNA Kit (STRATEC Molecular GmbH, Birkenfeld, Germany) according to the manufacturer’s instructions and quantified with the QUBIT dsDNA Assay Kit (Life Technologies, Carlsbad, CA, USA). DNA was diluted to a final concentration of 3 µg/mL.

The Ion 16S™ Metagenomics Kit (Life Technologies, Carlsbad, CA, USA) was used to amplify V2-4-8 and V3-6, V7-9 regions of the 16S rRNA gene in separate PCR reactions. Amplicons were purified using Agencourt^®^ AMPure^®^ XP beads (Beckman Coulter, Brea, CA, USA), quantified on a LabChip^®^ GX Touch™ system (PerkinElmer, Waltham, MA, USA), and prepared for sequencing with the Ion Plus Fragment Library Kit and Ion Xpress™ barcode adapters (both Thermo Fisher Scientific, Waltham, MA, USA). Library preparation was automated on the Ion Chef™ instrument using Ion 510™, 520™, and 530™ Chef Kits and loaded onto an Ion 350™ chip (all Thermo Fisher Scientific, Waltham, MA, USA). Sequencing was performed on an Ion S5™ system (Thermo Fisher Scientific, Waltham, MA, USA).

To minimize potential batch effects, all samples were processed using the same DNA extraction kit, library preparation protocol, and Ion Torrent^TM^ sequencing workflow. Samples were multiplexed and sequenced within a single run, ensuring uniform reagent and instrument conditions. Bioinformatic processing (described below), including quality filtering and taxonomic assignment, was carried out using the same Ion Reporter^TM^ Software v.5.10 for all samples. Technical sequencing replicates were not performed.

### 2.5. Bioinformatics and Diversity Analysis

Raw reads were processed with Ion Reporter™ Software v5.10 (Thermo Fisher Scientific, Waltham, MA, USA), which incorporates QIIME bioinformatics tools [28]. Primer sequences and reads <150 bp were removed. Sequences were aligned against the Curated MicroSEQ^®^ 16S Reference Library v2013.1 with a minimum of 90% alignment coverage. Sequences were clustered into genus-level Operational Taxonomic Units (OTUs) at 97% identity. OTUs present in <50% of samples per group or with relative abundance <0.1% were excluded. Alpha diversity indices (Chao1, Shannon, Simpson) and beta diversity (Bray—Curtis’s dissimilarity) were calculated. Principal coordinates analysis (PCoA) and generation of heatmaps were performed using the Vegan Community Ecology package https://CRAN.R-project.org/package=vegan, accessed on 27 January 2025) and Bioconductor [29] in R (v3.6.0). A Venn diagram was generated using Venny2.1.0 [30]. Differential abundance analysis was performed using LEfSe [31] as implemented in Galaxy server (http://galaxy.biobakery.org/, accessed on 27 January 2025) with a linear discriminant analysis (LDA) cutoff of >2.5. Correlation analyses used Spearman’s rank coefficients and partial correlations adjusted for pre-pregnancy BMI and GWG. The red color of a square indicates a positive correlation between the two variables, and the blue color indicates a negative correlation. Statistically significant correlations (*p* ≤ 0.05) between variables are marked with an asterisk.

### 2.6. Statistical Analysis

All statistical analyses were performed with IBM SPSS Statistics 26 (IBM Corp., Armonk, New York, NY, USA). Normally distributed variables were expressed as mean ± standard deviation and compared using independent-samples *t*-tests. Non-normally distributed variables were expressed as median (interquartile range) and compared using Mann–Whitney U tests. *p*-values < 0.05 after false discovery rate (FDR) correction were considered statistically significant.

## 3. Results

### 3.1. Characteristics of the Study Population

The characteristics of the study population are summarized in Table 1. Altogether, 14 parameters were collected. Based on infant birth weight, participants were categorized into two groups: the LGA group (*n* = 8) and the NBW group (*n* = 10).

Maternal pre-pregnancy weight and BMI were significantly higher in the LGA group compared with the NBW group (*p* = 0.041 and *p* = 0.016, respectively). Both the LGA and NBW groups include mothers meeting criteria for obesity. The LGA group showed a significantly higher HOMA-IR index than the NBW group (*p* = 0.034). GWG was lower in the LGA group, but this difference was not statistically significant (*p* = 0.100). All participants reported adherence to dietary recommendations and maintained good glycemic control [27], HbA1c values did not differ significantly between groups in either the 2T or 3T (*p* = 0.961 and *p* = 0.529, respectively). Interestingly, hemoglobin concentration in the 3T was lower in the LGA group (*p* = 0.018).

### 3.2. Estimates of Microbial Diversity

**Figure 1:**
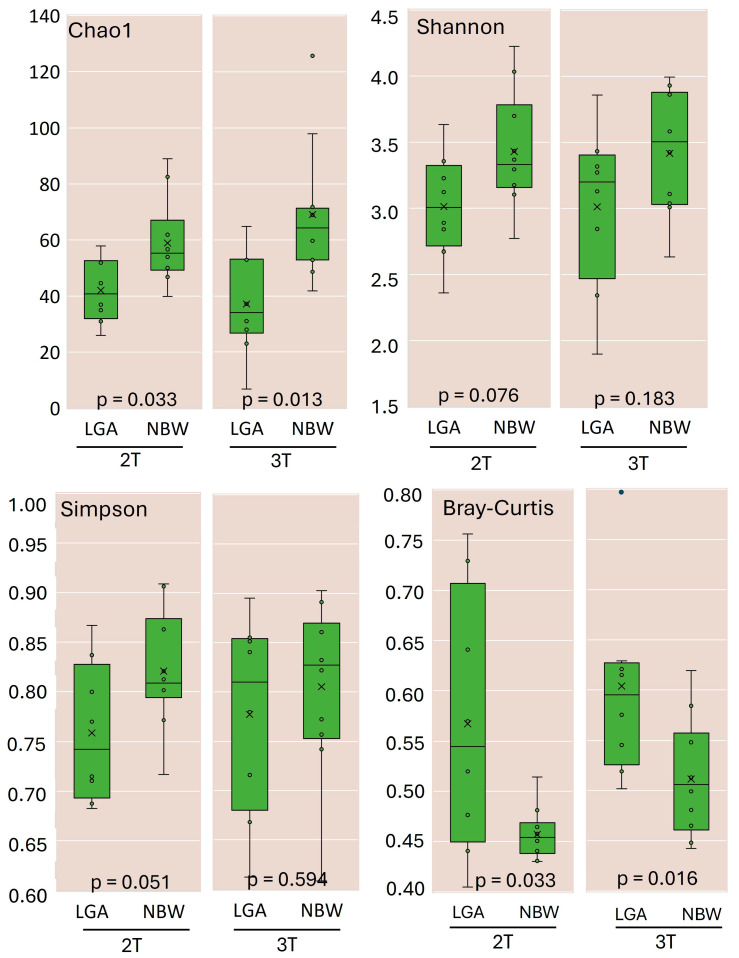
Richness and diversity of gut microbiota in the LGA and NBW groups during the 2T and 3T trimesters of pregnancy. Alpha-diversity indices, including the Chao1 estimator, Shannon index, and Simpson index, were calculated to assess the richness and diversity of the gut microbiota in the NBW and LGA groups. Beta diversity was evaluated using the Bray–Curtis dissimilarity index. All diversity metrics were calculated at 97% sequence similarity using QIIME 2 software, as implemented within the Ion Reporter^TM^ platform. x—mean value, ◦—individual observation.

Twenty women were excluded from this study for various reasons, and one was excluded due to insufficient sequencing depth of the collected samples. This left a total of 36 samples. This dataset contained 10,435,083 valid reads. Upon quality filtering, the mean sequencing depth was 108,589 reads (range 26,925–203,936). All downstream diversity analyses were performed on OTU tables rarefied to 25,000 reads per sample, retaining all remaining samples. Alpha diversity analysis (Chao1, Shannon, and Simpson indices) (Figure 1) revealed that the LGA group exhibited significantly reduced microbial richness and lower overall diversity at both 2T and 3T compared with the NBW group.

Diversity indices increased over time in both groups, indicating an increase in richness commonly observed as pregnancy progresses. Beta diversity (Bray—Curtis’s dissimilarity index, Figure 1) also increased in both groups but remained significantly higher in the LGA group across trimesters, reflecting higher between-subject variability. Principal coordinates analysis (PCoA) did not reveal distinct clustering by group or trimester (Supplementary Figure S1).

### 3.3. Variations in Maternal Gut Microbiota in GDM Pregnancies According to Infant Birth Weight and Gestational Trimester

**Figure 2:**
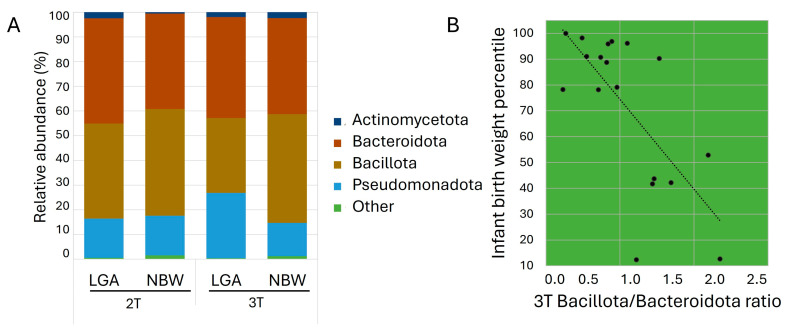
(**A**) Average relative abundance of bacterial taxa at the phylum level in the study groups. Phyla with relative abundance <0.1% are grouped as ‘Other’; (**B**) Scatter plot showing the relationship between infant birth weight percentile and the Bacillota/Bacteroidota ratio in the 3T.

At the phylum level, Bacillota and Bacteroidota dominated, followed by Pseudomonadota and Actinomycetota (Figure 2A). In the 2T, the LGA group demonstrated a lower relative abundance of Bacillota (38.5% vs. 43.2%) and higher abundances of Bacteroidota (42.6% vs. 38.6%) and Actinomycetota (2.4% vs. 0.6%) compared with the NBW group. By the 3T, the LGA group showed a significant increase in Pseudomonadota (26.5%, *p* < 0.021) and a marked decrease in Bacillota (13.5%, *p* < 0.021). The Bacillota/Bacteroidota ratio decreased from 1.09 in the 2T to 0.80 in the 3T in the LGA group, while it remained stable at 1.12 in the NBW group. This ratio inversely correlated with infant birth percentile (*r* = −0.622, *p* < 0.01) (Figure 2B), and linear regression confirmed that a higher Bacillota/Bacteroidota ratio predicted lower infant birth weight (R^2^ = 0.472, *p* < 0.001; β = −0.709, 95% CI: −53.1 to −16.5, *p* = 0.016). No additional significant phylum-level differences were observed between trimesters in either group, indicating that the overall gut microbiota composition remained relatively stable throughout this period (Supplementary Figure S2A).

**Figure 3:**
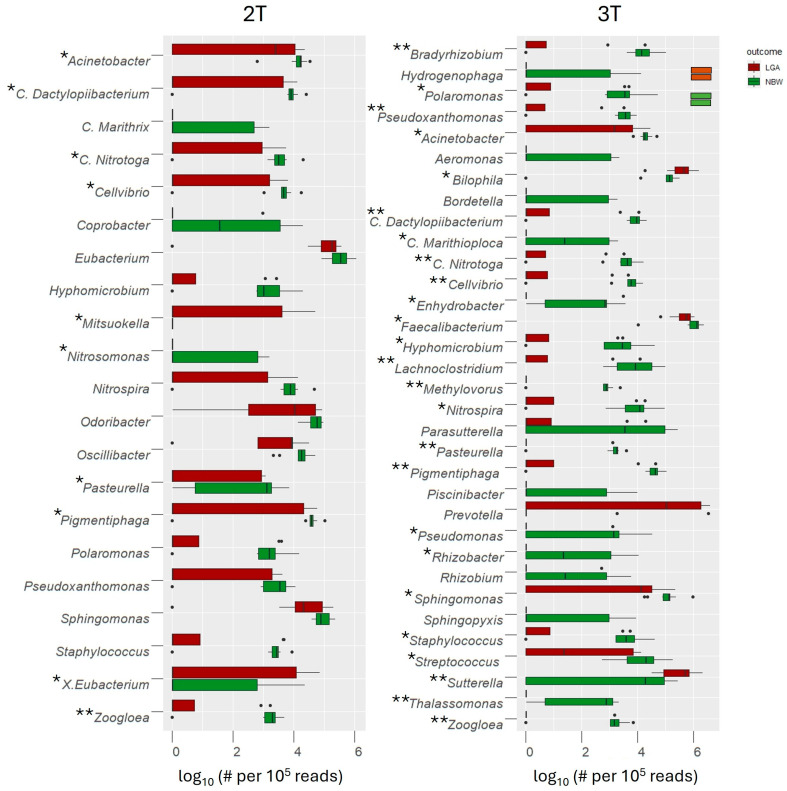
Genera with significantly different abundances between the LGA and NBW groups were identified using the Mann–Whitney U test, with false discovery rate (FDR) correction applied for multiple comparisons (** *p* < 0.01—highly significant; * *p*  <  0.05—significant, *p* < 0.1—trend). Genus-level abundance in each sample was normalized to 100,000 reads and log_10_-transformed to facilitate visualization. #—number, dots represent individual sample abundances for each taxon within the LGA and NBW groups, plotted with horizontal jitter to illustrate the underlying distribution of log_10_-transformed read counts.

Among the 227 genus-level OTUs identified, 78 (37.7%) were shared across all groups (Supplementary Figure S2B). Further analysis revealed clear between-group differences. In the 2T, 21 OTUs differed significantly between groups; 19 were depleted in the LGA group, including commensal taxa such as *Acinetobacter*, *Coprobacter*, *Staphylococcus*, and *Odoribacter*, while *Mitsoukella* was enriched. By the 3T, 33 OTUs showed differential abundance, again with notable depletion of beneficial taxa (e.g., *Faecalibacterium*, *Odoribacter*, *Lachnoclostridium*) and enrichment of pro-inflammatory or opportunistic genera such as *Prevotella*, *Sutterella*, and *Bilophila*. *Acinetobacter* remained consistently depleted in the LGA group throughout. Interestingly, while *Eubacterium* was depleted in mothers delivering LGA infants, the subgroup *X. Eubacterium* was enriched. All observations were supported by statistical analyses at the genus level OTUs, with key groups presented in Figure 3. OTUs with *p*-values < 0.01 were regarded as highly statistically significant (**), with values between 0.01 and 0.05 as statistically significant (*), while OTUs with *p*-values between 0.051 and 0.1 were interpreted as trends.

**Figure 4:**
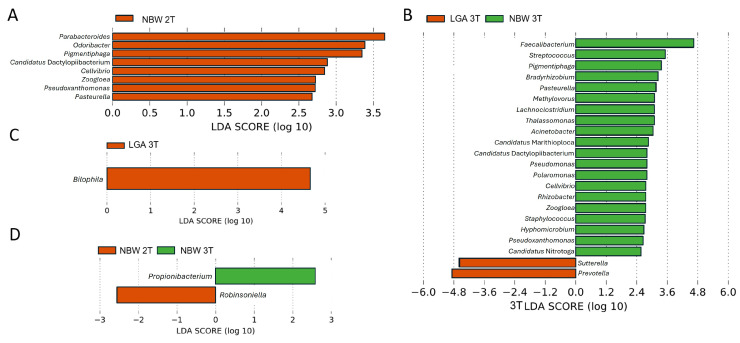
Differentially abundant genera in NBW and LGA GDM groups identified by LEfSe analysis. The LDA score (log_10_) indicates the effect size of each taxon (**A**) Genera enriched during the 2T (note that only NBW group contained enriched taxa), (**B**) Genera enriched during the 3T, (**C**) genera enriched in LGA group during the transition from the 2T to the 3T, (**D**) genera enriched in the NBW group during the transition from the 2T to the 3T.

LEfSe analysis (LDA score > 2.5) confirmed these trends. Genera enriched in the 2T, in the NBW group included SCFAs producers and beneficial commensals *Parabacteroides*, *Odoribacter,* as well as *Pigmentiphaga*, *Candidatus* Dactylopiibacterium, *Cellvibrio*, *Zoogloea*, *Pseudoxanthomonas*, and *Pasteurella* (Figure 4A). By the 3T, NBW pregnancies showed stronger enrichment of 20 beneficial taxa (e.g., *Faecalibacterium*, *Lachnoclostridium*, *Acinetobacter*), while LGA pregnancies were characterized by enrichment of *Sutterella* and *Prevotella* genera previously linked to inflammation and altered metabolic regulation (Figure 4B). *Bilophila*, a known pro-inflammatory bacterium, became enriched during the 2T to 3T transition in LGA pregnancies (Figure 4C).

In the NBW group, temporal differences were also observed, with *Propionibacterium* (now *Cutibacterium*) enriched in the 2T and *Robinsoniella* in the 3T (Figure 4D). These findings again suggest that protective, SCFAs-producing bacteria are comparatively enriched in NBW GDM pregnancies, whereas pro-inflammatory taxa are enriched in LGA, particularly as pregnancy progresses (3T).

### 3.4. Gut Microbiota of Mothers with GDM Differs with Respect to the Pre-Pregnancy Body Mass Index (BMI), Gestational Weight Gain (GWG), and Infant Birth Weight

**Figure 5:**
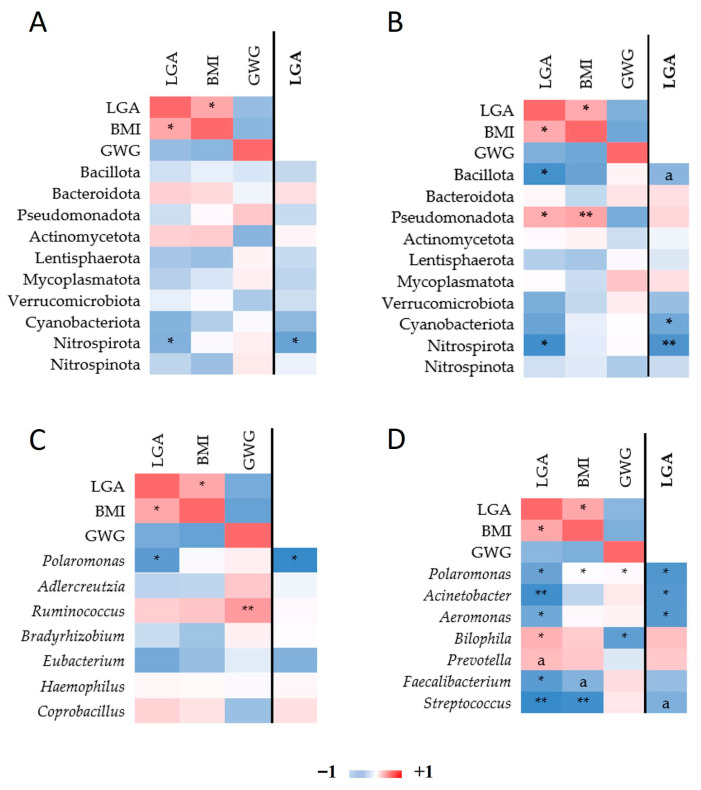
Heatmap of Spearman correlations among bacterial taxa and pre-pregnancy BMI, GWG, and LGA with respect to pregnancy trimester. Spearman’s rho among phylum read counts, LGA, BMI, and GWG in the 2T (**A**) and in the 3T (**B**). Correlation among genus read count, LGA, BMI, and GWG in the 2T (**C**) and in the 3T (**D**). In the columns on the right (distinguished with the line; LGA marked in bold font), the correlations among read counts and LGA are adjusted by BMI and GWG. Only taxa nominally associated with either of the three traits are depicted. * *p*  ≤  0.05, ** *p*  ≤  0.01. Mark “a” designates trend *p* ≤  0.1.

After adjusting for pre-pregnancy BMI and GWG, several taxa retained significant associations with infant birth weight. At the phylum level, Nitrospirota correlated negatively with LGA births in both trimesters, with stronger significance in the 3T. Bacillota also correlated negatively with LGA in the 3T, while Cyanobacteriota did so only after adjustment. Pseudomonadota initially correlated positively with LGA and BMI but lost significance after adjustment (Figure 5A,B).

At the genus level (Figure 5C,D), *Polaromonas* correlated negatively with LGA in both trimesters. In the 3T, *Acinetobacter* and *Aeromonas* showed negative correlations with LGA after adjustment. *Bilophila* was initially positively correlated with LGA and identified as a LEfSe biomarker; however, this association was no longer significant after adjusting for maternal BMI and GWG.

## 4. Discussion

### 4.1. Lower Maternal Gut Microbial Diversity and Phylum-Level Shifts in GDM May Contribute to LGA Risk

Our findings addressed whether variations in the maternal microbiota among women with GDM were associated with the delivery of a LGA infant. Early studies have shown reduced microbial diversity in women with GDM compared to normoglycemic pregnancies [18,21]. However, evidence remains inconsistent, with other studies reporting increased maternal gut microbial diversity in GDM pregnancies [21,24,32], highlighting the need for further investigation.

In our study, both alpha- and beta-diversity increased from the 2T to the 3T, as reported in some studies [21,24]. Such shifts are characteristic of the microbiota remodelling that occurs in late pregnancy to meet the evolving metabolic, endocrine, and immunological demands of gestation. However, a comparison between the two groups studied showed that although the overall diversity increased along the pregnancy progress, the LGA group exhibited significantly lower species richness and diversity, along with greater inter-individual variation, indicating an additional layer of dysbiosis. This reduced diversity in the LGA group may mirror altered metabolic states previously linked to GDM.

At the phylum level, our findings confirm and extend prior reports linking GDM to characteristic shifts in gut microbial composition [23,24,33]. In the NBW group, pregnancy progression from the 2T to the 3T was marked by a decline in Pseudomonadota and an increase in Bacillota. In contrast, the LGA group exhibited more pronounced alterations. By the 3T, women who delivered LGA infants showed reduced abundances of Bacteroidota, Bacillota, and Actinomycetota, coupled with an expansion of Pseudomonadota and a marked decrease in the Bacillota/Bacteroidota ratio. This reduced ratio was significantly associated with LGA. Such phylum-level shifts have been linked to impaired energy harvest, diminished SCFAs production, and increased lipopolysaccharide (LPS) biosynthesis—mechanisms that can drive systemic inflammation, exacerbate insulin resistance, and elevate fetal glucose exposure [34,35,36]. All this could contribute to LGA risk.

### 4.2. LGA Pregnancies Are Associated with Depletion of Protective SCFAs-Producing Genera and an Enrichment of Pro-Inflammatory Taxa

Genus-level OTU analysis revealed differences in taxonomic abundance between the LGA and NBW groups, characterized by significant depletion or complete loss of specific taxa in the LGA group. These taxa comprised environmental bacteria, likely representing transient members of the gut microbiome, as well as several candidate genera whose functional roles remain largely uncharacterized. Notably, several well-established genera, particularly SCFAs producers, were markedly depleted.

The abundance of the genus *Acinetobacter*, previously reported to be reduced in GDM pregnancies and negatively correlated with inflammation, insulin resistance, and impaired glucose tolerance [37], was significantly lower in the LGA group at 2T and declined further by 3T. In these terms, this reduction is consistent with the progressive increase in insulin resistance observed during pregnancy [38].

At 2T, we observed a trend toward reduced abundance of *Odoribacter*, potentially reflecting a diminished capacity for metabolic regulation. *Odoribacter laneus*, consumes succinate and contributes to metabolic homeostasis through the succinate–SUCNR1 signalling axis [39]. The reduction in abundance of this bacterium increases circulating succinate levels, aggravating gut dysbiosis in GDM and driving metabolic disturbances seen in Type 2 diabetes and obesity [39]. Consistent with our findings, Serena et al. [40] observed a similar depletion of *Odoribacter* in individuals with obesity, despite the functional role of this genus in the gut microbiome remaining poorly defined.

Conversely, *Mitsuokella* was overrepresented in the LGA group. Its production of trimethylamine, a metabolite linked to cardiometabolic disorders, including type 2 diabetes [41] may disrupt maternal metabolic homeostasis and contribute to excessive fetal growth.

At 3T, the microbiota of mothers with LGA infants showed further depletion of the SCFAs-producing genus *Acinetobacter* and other key taxa, including *Faecalibacterium* and *Lachnoclostridium*. Depletion of *Faecalibacterium* appears to be a hallmark of GDM pregnancies [42], positioning this genus as a “sentinel of the gut” and a potential biomarker of maternal metabolic health. Regarding *Lachnoclostridium*, Shen et al. [43] reported a decline in its abundance during normal pregnancy, with a decrease in abundance in women with GDM, suggesting that appropriate modulation of this genus may confer metabolic benefits.

At 3T, *Prevotella*, *Sutterella*, and *Bilophila* were significantly overrepresented in the LGA group and were identified as taxonomic biomarkers by LEfSe. All three genera have been associated with elevated inflammation, impaired glucose tolerance, and increased insulin resistance, directly or indirectly, indicating potential roles in the metabolic disturbances observed in mothers of LGA infants [44,45,46].

Ferrocino et al. [24] reported that *Prevotella* was significantly associated with HbA1c levels and correlated with mucin oligosaccharide degradation, contributing to gut permeability dysfunction. Wu et al. [45] causally linked elevated *Prevotella* levels to increased GDM risk across all trimesters of pregnancy. In addition, *Sutterella* showed a direct association with C-reactive protein levels and a positive correlation with LPS biosynthesis pathways in multiple regression models [24,45].

*Bilophila* showed increased abundance in the LGA group. This genus, represented primarily by *B. wadsworthia*, is closely linked to bile acid metabolism and is known to proliferate in individuals with metabolic syndrome. Its expansion is strongly associated with diets rich in animal fats, which increase taurocholic acid—a bile salt that promotes the growth of this sulfite-reducing bacterium [47]. Furthermore, Wu et al. [48] identified *Bilophila* among key bacterial genera significantly enriched in GDM using multivariate discriminant analysis [48]. Consistently, our LEfSe analysis identified *Bilophila* as a candidate biomarker of adverse pregnancy outcomes, specifically in the 3T of LGA pregnancies.

### 4.3. The Critical Role of Pre-Pregnancy BMI and GWG

Understanding the relationship between gut microbiota and LGA outcomes requires careful consideration of maternal metabolic factors. Women who gave birth to LGA infants demonstrated significantly higher pre-pregnancy BMI. Furthermore, they gained less weight during pregnancy or even lost weight compared to women with NBW infants. We hypothesize that pre-existing maternal adiposity may provide sufficient metabolic reserves to support excessive fetal growth, reducing the reliance on additional GWG. This raises the possibility that pre-pregnancy BMI shapes maternal gut microbiota in ways that influence fetal growth, suggesting a mechanistic link between maternal adiposity, microbial composition, and LGA risk.

After adjusting gut microbiota read counts for pre-pregnancy BMI and GWG, several taxa were significantly associated with LGA risk in Spearman correlation analyses. Nitrospirota was negatively correlated with LGA at 2T, which strengthened in 3T following BMI and GWG adjustment. *Polaromonas,* after adjustment, also showed consistent negative correlations with LGA in both trimesters. Given that Nitrospirota and *Polaromonas* members are primarily environmental bacteria, we hypothesize that they colonize the gut transiently or in low abundance. Although low-abundance, transient taxa may influence local metabolic processes and contribute to dysbiosis [49]. Nitrospirota could affect nitrogen cycling [50,51] and *Polaromonas,* the degradation of organic compounds [52] and could serve as minor contributors or proxy markers linked to host metabolic phenotypes [53]. Their precise functional roles in the maternal gut and potential impact on fetal growth remain to be elucidated.

In the 3T, *Acinetobacter* and *Aeromonas* abundances negatively correlated with LGA after adjustment for BMI and GWG. These findings reinforce the protective role of *Acinetobacter* and highlight *Aeromonas* as another potentially beneficial genus in late pregnancy.

Correlation analyses further confirmed the positive association between *Bilophila* abundance and LGA in the 3T. However, after adjustment for maternal BMI and GWG, the association between *Bilophila* and LGA was no longer significant, suggesting that its link with LGA is mediated by maternal adiposity rather than representing an independent driver. Thus, *Bilophila* may serve as an indicator of maternal metabolic status rather than as a direct contributor to excessive fetal growth.

### 4.4. Study Limitations

Among the initial cohort of 39 recruited women, 18 met the inclusion criteria after applying the exclusion criteria. Hence, this study’s final sample size limits its statistical power to detect subtle differences in gut microbiota and constrains the generalizability of the findings. The cross-sectional design, based on two discrete time points, provides only a partial view of the dynamic changes in gut microbiota throughout pregnancy. Furthermore, because fecal microbiota composition can be influenced by short-term dietary fluctuations, future studies should complement this design with detailed pre-sampling dietary records to enhance interpretation of transient microbial signatures

A key limitation of this study is the lack of a normoglycemic pregnancy control group. This substantially limits the interpretation of our findings, as it prevents clear differentiation between microbiota characteristics attributable to GDM and those specifically associated with LGA outcomes. Consequently, the observed microbial shifts may represent a combined signature of maternal hyperglycemia and fetal overgrowth rather than GDM alone. We explicitly acknowledge this constraint and emphasize that future research incorporating normoglycemic pregnant women with matched gestational age and neonatal characteristics will be essential for disentangling these effects.

In addition, elevated pre-pregnancy BMI in the LGA group represents a significant confounding factor, as it may independently affect both microbiota composition and fetal development. The complex interplay among maternal BMI, GWG, and gut microbial profiles warrants further investigation to disentangle causal relationships. The lack of metabolomic data limits this study’s ability to provide functional validation for the proposed mechanistic pathways linking microbiota alterations to fetal growth trajectories.

Finally, this study is monocentric, with participants originating from a limited geographical area. Although dietary habits in this population may be diverse, partial alignment of dietary patterns is expected due to standardized nutritional guidance provided after a GDM diagnosis. Consequently, the generalizability of our findings to populations from different regions, ethnic backgrounds, or with differing dietary recommendations may be limited.

## 5. Conclusions

This pilot longitudinal study provides evidence that specific maternal gut microbiota profiles are associated with LGA birth outcomes in GDM pregnancies, independent of glycemic control. Mothers of LGA infants exhibited lower alpha and beta diversity, a reduced Bacillota/Bacteroidota ratio, enrichment of pro-inflammatory taxa (*Prevotella*, *Sutterella*, *Bilophila*), and depletion of SCFAs-producing genera (*Odoribacter*, *Faecalibacterium*, *Lachnoclostridium*). Among these taxa, *Bilophila* was identified as a 3T biomarker. However, its association with LGA was no longer significant after adjusting for pre-pregnancy BMI and GWG, indicating that it may reflect maternal adiposity rather than directly drive fetal overgrowth. Negative correlations of Nitrospirota, *Polaromonas*, *Acinetobacter*, and *Aeromonas* with LGA persisted even after adjustment, suggesting potentially protective roles. These results highlight complex interactions between maternal adiposity, microbial community structure, and SCFAs metabolism that shape fetal growth. The identification of microbial signatures months before delivery offers a foundation for future biomarker development and microbiota-targeted interventions to reduce the risk of LGA in GDM.

## Figures and Tables

**Table 1:** Characteristics of the study population.

Parameter	Altogether N = 18	LGA-Infants N = 8	NBW-Infants N = 10	*p*-Value
Age (years)	33 (4)	35 (4)	31 (4)	0.108
Body Mass Index (kg/m^2^)	23.5 (21.5; 28.8)	28.1 (5.8)	22.0 (21.2; 25.0)	0.016
Gestational weight gain (kg)	9.5 (5.1)	6.3 (3.9; 8.8)	11.1 (4.7)	0.100
Fasting glucose, 2T (mmol/L)	4.9 (0.5)	5.1 (0.5)	4.7 (0.6)	0.111
HbA1c, 2T (%)	5.0 (0.3)	5.0 (0.4)	5.0 (0.2)	0.961
HbA1c, 3T (%)	5.2 (0.4)	5.0 (0.3)	5.2 (0.3)	0.529
HOMA-IR index	2 (1.3; 3.6)	2.6 (2.1; 5.3)	1.3 (0.9; 3.2)	0.034
Total cholesterol (mmol/L)	6.6 (0.9)	6.2 (0.7)	6.8 (1.0)	0.254
HDL-cholesterol (mmol/L)	2.1 (0.4)	2.0 (0.4)	2.1 (0.4)	0.663
LDL-cholesterol (mmol/L)	3.7 (0.9)	3.3 (0.4)	3.9 (1.0)	0.072
Triglycerides (mmol/L)	2.0 (0.5)	2.1 (0.6)	2.0 (0.5)	0.870
Alanine aminotransferase (µkat/L)	0.24 (0.20; 0.27)	0.24 (0.08)	0.25 (0.20; 0.31)	0.397
Hemoglobin concentration, 2T (g/L)	128.8 (7.6)	127.8 (7.2)	129.7 (8.3)	0.630
Hemoglobin concentration, 3T (g/L)	117.0 (8.3)	112.9 (9.1)	121.8 (4.4)	0.018

Normally distributed continuous variables were reported as mean ± standard deviation, while non-normally distributed continuous variables were reported as median with interquartile ranges (Q1–Q3).

## Data Availability

The data presented in this study are openly available in the NCBI Sequence Read Archive under the BioProject accession number PRJNA1031979, corresponding to BioSample numbers SAMN37977233-SAMN37977268.

## Supplementary Materials

The following supporting information can be downloaded at: https://www.mdpi.com/article/10.3390/biomedicines13122941/s1, Figure S1: Principal coordinates analysis (PCoA)—clustering by group or trimester (LGA 2T, LGA 3T, NBW 2T, NBW 3T) based on Bray-Curtis distances; Figure S2: (A) A heatmap of the relative abundance of bacterial taxa at the phylum level across individual samples, grouped by trimester and birth weight category. (B) Venn diagram showing the overlap of genus-level OTUs among individual samples.

## Abbreviations

The following abbreviations are used in this manuscript:

2T	Second trimester
3T	Third trimesters
BMI	Body mass index
FDR	False discovery rate
GDM	Gestational diabetes mellitus
GVG	Gestational weight gain
LDA	Linear discriminant analysis
LGA	Large-for-gestational age
LPS	Lipopolysaccharide
NBW	Normal birth weight
SCFAs	Short-chain fatty acids
OTU	Operational taxonomic unit
PCoA	Principal coordinates analysis
