# Supplementary material for: Maternal Gut Microbiota in Gestational Diabetes Mellitus and Fetal Macrosomia: Is There an Association?
Source: Biomedicines. 2025 Nov 29;13(12):2941. doi: 10.3390/biomedicines13122941 (PMC12731141; doi:10.3390/biomedicines13122941)
Supplement: Supplementary file 1 [file biomedicines-13-02941-s001.zip › biomedicines-3993658-supplementary.pdf]

Supplementary material

Maternal Gut Microbiota in Gestational Diabetes Mellitus and Fetal Macrosomia: Is there an Association?

Lejla Pašić, Katja Molan, Draženka Pongrac Barlovič, Marjanca Starčič Erjavec, Darja Žgur Bertok, Jerneja Ambrožič Avguštin

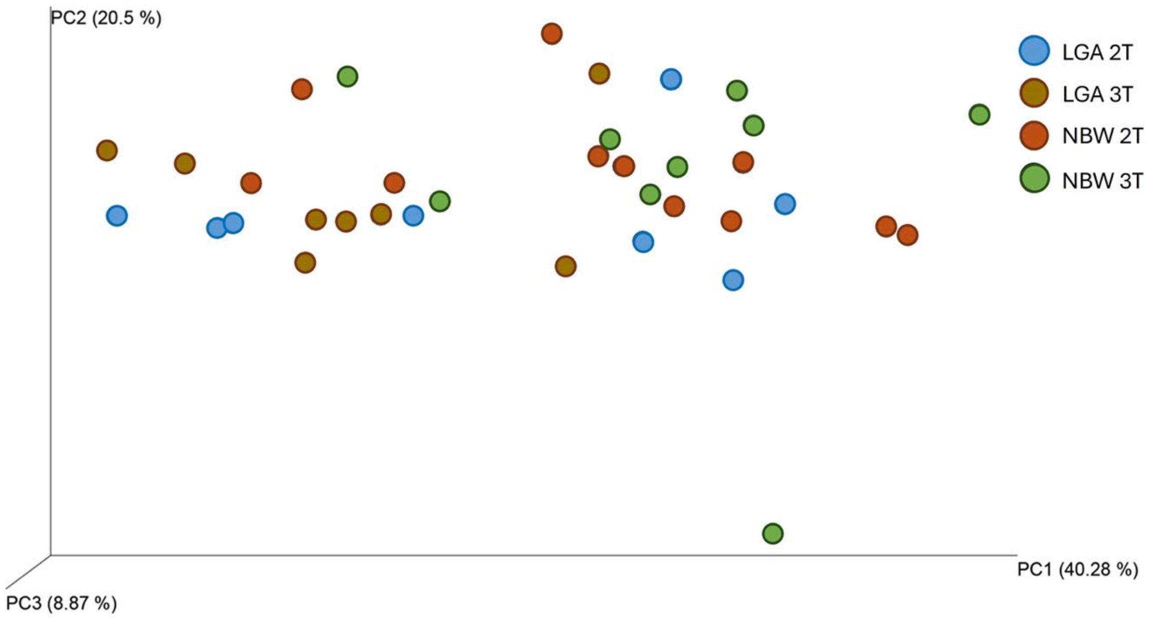

Supplementary Figure S1. Principal coordinates analysis (PCoA) - clustering by group or trimester (LGA 2T, LGA 3T, NBW 2T, NBW 3T) based on Bray-Curtis distances.

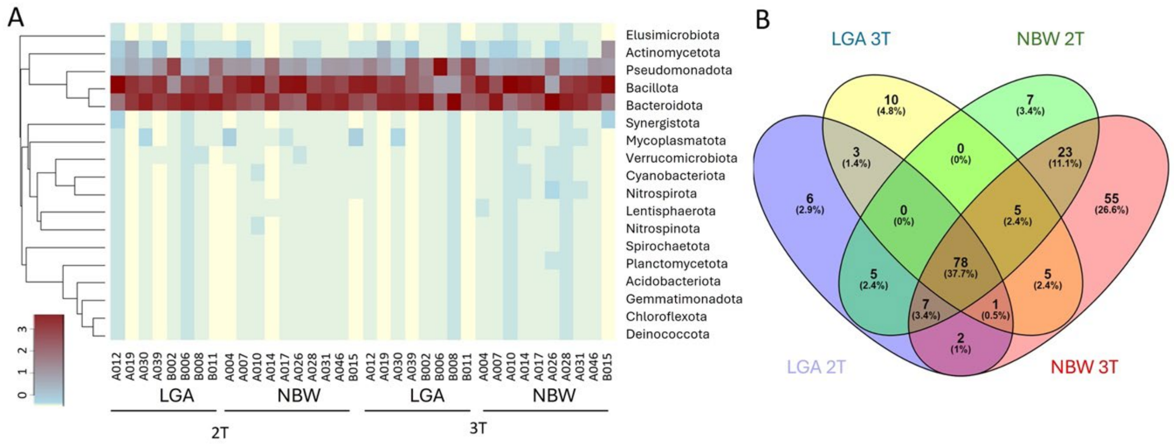

Supplementary Figure S2. A) A heatmap of the relative abundance of bacterial taxa at the phylum level across individual samples, grouped by trimester and birth weight category. B) Venn diagram showing the overlap of genus-level OTUs among individual samples. 2T- second trimester; 3T- third trimester; NBW - normal birth weight group, LGA – large-for-gestational-age group.
